# On the initiation of lightning in thunderclouds

**DOI:** 10.1038/s41598-017-01288-0

**Published:** 2017-05-02

**Authors:** Ashot Chilingarian, Suren Chilingaryan, Tigran Karapetyan, Lev Kozliner, Yeghia Khanikyants, Gagik Hovsepyan, David Pokhsraryan, Suren Soghomonyan

**Affiliations:** 1 Yerevan Physics Institute, 2 Alikhanyan Brothers, 0036 Yerevan, Armenia; 2 National Research Nuclear University MEPhI (Moscow Engineering Physics Institute), Moscow, 115409 Russian Federation

## Abstract

The relationship of lightning and elementary particle fluxes in the thunderclouds is not fully understood to date. Using the particle beams (the so-called Thunderstorm Ground Enhancements – TGEs) as a probe we investigate the characteristics of the interrelated atmospheric processes. The well-known effect of the TGE dynamics is the abrupt termination of the particle flux by the lightning flash. With new precise electronics, we can see that particle flux decline occurred simultaneously with the rearranging of the charge centers in the cloud. The analysis of the TGE energy spectra before and after the lightning demonstrates that the high-energy part of the TGE energy spectra disappeared just after lightning. The decline of particle flux coincides on millisecond time scale with first atmospheric discharges and we can conclude that Relativistic Runaway Electron Avalanches (RREA) in the thundercloud assist initiation of the negative cloud to ground lightning. Thus, RREA can provide enough ionization to play a significant role in the unleashing of the lightning flash.

## Introduction

Among top unanswered questions in lightning research^1^ state as number one: “*By what physical mechanism or mechanisms is lightning initiated in the thundercloud*?” and - number two: “*What physical mechanisms govern the propagation of the different types of lightning leaders*?”.

They also mentioned that “*The problem of how lightning is initiated inside thunderclouds is not only one of the biggest unsolved problems in lightning physics; it is also probably one of the biggest mysteries in the atmospheric sciences*”.

One of the candidates related to initiation and propagation of lightning is considered to be energetic runaway electrons. Electron acceleration in the thunderstorm atmospheres was first recognized by CTR Wilson^2^; then Gurevich *et al*.^3^ introduced the electron runaway concept (named Runaway Breakdown - RB, now mostly referred as Relativistic Electron Runaway Avalanche – RREA); in 2003 J. Dwyer^4^ developed the feedback model of intracloud electron-gamma ray avalanches exponentially enhancing electron number. Recent observations of hundreds of the Thunderstorm ground enhancements (TGE, an abrupt enhancement of the secondary cosmic rays measured on the Earth’s surface in correlation with thunderstorms) on Aragats provide an extensive source for the development of models of particle acceleration and multiplication in thunderclouds^5, 6^.

The electric field strength and spatial extent required for the RB/RREA development was measured during balloon flights in thunderstorm atmospheres at New Mexico. A 1.87 kV/cm field extended 1 km downwards from the height of 5.77 km would give an RREA multiplication factor of about 650^7^. *In situ* measurements of the RREA by the network of particle detectors on Aragats allow retrieving the RREA parameters and developing a TGF initiation model^8, 9^. Estimated multiplication factor was ~330, e-folding length ~250–300 m and maximum energy of RREA electrons in the cloud - 40–50 MeV. The strength of the uniform vertically downward field of 1.5 km elongation expected to be 1.8–2.0 kV/cm. For the both observed cases, RREA electron flux will significantly increase the electrical conductivity in the cloud and possibly would not only introduce an additional leakage current but also can assist propagation of the lightning leader.

In this study, we analyze a special kind of TGEs, i.e. TGEs abruptly terminated by lightning flashes. To our knowledge, first reports on the particle flux abruptly terminated by lightning flash come from measurements made on board of NASA STORM Hazard Project F-106 aircraft. The X-ray flux is sometimes seen to increase prior to observed lightning discharge and then return to background level^10^. The balloon flights near Norman, Oklahoma in the spring of 1995 reveals an increase in X-ray intensity of 2 orders of magnitude lasting for approximately 1 min. The X-ray intensity returned to background level at the time of a lightning flash that reduced the electric field strength measured at the balloon^11^. The Baksan group reported the first TGEs of this kind^12^. They demonstrated that the particle count rate increased at energies of ~30 MeV then quickly returned to the background level when lightning occurred. In^13^ they deduce that the lightning flashes serve as a switch-off for the electric field. Recently several groups report such special TGEs as well^14–18^.

Thus, using additional key observables, the TGEs, we investigate relations between RREA propagation in the cloud and occurrence of nearby lightning flashes and gain insights into the role of energetic runaway electrons in lightning initiation.

The main method of the multivariate data analysis and physical inference consists in the selection of the hierarchical time series of particle count rates along with measurements of the electrostatic electric field, distance to lightning, fast electric field waveforms and other. Precise synchronization of all measurements allows analyzing the time series on millisecond time scales. The one-second and one-minute time series are very useful for discovering many non-trivial correlations in TGE data. Analyzing numerous TGEs with one and the same sequence of patterns we reveal the repeating structures, typical correlations and finally causal relations between observables. Multivariate analysis methodology has been made possible by the use of Advanced Data Extraction Infrastructure - a very flexible and powerful tool providing services for the multidimensional visualization, data zooming, comparison, digitizing, statistic analysis and other.

## Methods: registration of the TGEs and associated geophysical parameters by the networks of particle detectors and field-meters

The particle detectors of the Aragats Space Environmental Center (ASEC^19^) measure the fluxes of the neutral and charged species of secondary cosmic rays. Numerous thunderstorm-correlated events, detected by the ASEC facilities, constitute a rich experimental set for the investigation of the high-energy phenomena in the thunderstorm atmosphere. The new generation of ASEC detectors consists of 1- and 3-cm-thick molded plastic scintillators arranged in stacks (named STAND1 and STAND3) and cubic structures (named CUBE1 and CUBE3); see the appendix. A detailed description of ASEC detectors, including charts with all sizes, is available from the WEB site of the Cosmic Ray Division of Yerevan Physics Institute http://crd.yerphi.am/ADEI in the WIKI section of the multivariate visualization platform and from^5, 20^. With networks of these and other operated on Aragats particle detectors, we continuously monitor incident particle fluxes and geophysical parameters. The data on particle fluxes is integrated and stored as 1-minute, 1-second and 50 ms time series of particle counts (number of particles detected each minute, each second, each 50 ms). Measurements of the electric field are performed with frequency 20 Hz; geomagnetic field and meteorological parameters – once a minute; cameras operate with frequency 30 Hz only when electric field strength exceeds a threshold value. When amplitude of the atmospheric discharges measured by an active whip antenna exceeds the threshold, the fast digital oscilloscope stores 1-second file with waveforms of atmospheric discharges. The data transfer from Aragats to Cosmic Ray Division (CRD) servers is performed each minute via fast radio-modems and is immediately assessable to users.

The detection efficiency of relativistic charged particle by plastic scintillator is ~99%. The detection efficiency of the neutral particle is proportional to the thickness of the scintillator, ranging from 1 to 20% for scintillators with the thickness 1–20 cm. Detectors are located outdoors or indoors under a minimal amount of matter allowing registration of low energy particles. The lowest energy threshold is ~0.4 MeV and ~1 MeV for neutral and charged particles, respectively.

The data acquisition (DAQ) system counts and stores all coincidences of the detector channel operation. For instance, the coincidence “100” of STAND1 detector denotes a signal in the upper detector only. This combination registered low-energy electrons with an efficiency of ~99%; for the outdoors location of STAND1 detector, the threshold energy is ~1 MeV. The gamma ray detection efficiency of this combination is 1–2%. The coincidence “010” selects mostly gamma rays as the probability to miss charge particle in the upper and bottom scintillators is ~0.01. The coincidence “111” means that all three layers register particles; the minimal energy of charged particles giving a signal in all three layers is above 10 MeV.

CUBE detector separates electron and gamma ray fluxes. The 1-cm thick scintillators surround two 20-cm-thick plastic scintillators. Both 20 cm thick and thin scintillators detect charged flux with a very high efficiency (~99%). Thick scintillators can also detect neutral flux with an efficiency of ~20%. The efficiency of detecting neutral flux by thin scintillators is below 2%. Thus, using the coincidences technique, it is possible to purify the neutral flux detected by inside scintillators, rejecting the charged flux by the veto signals from surrounding thin scintillators. The count rates of two inner thick scintillators and of the surrounding 6 scintillators are measured and stored each minute. In this way we estimate electron fraction in TGE each minute.

We recover the differential energy spectra of gamma-ray flux with the network of 7 NaI spectrometers. Each minute the histogram of energy releases in NaI crystals is stored and transferred to CRD database. During the off-line recovering of the energy spectra, we make energy-release to energy conversion using detector response simulation, taking into account bin-to-bin migration. We also measure energy release histograms with an assembly of four 60-cm thick and 1 m^2^ area plastic scintillators. The histograms of energy releases are stored each 20 sec that gives us possibility to precisely relate the lightning occurrence to the abrupt change of the TGE electron and gamma ray energy spectra.

With installing a new fast electronics at Aragats^21^ it became possible to simultaneously investigate time series of the near-surface electric field, fast electric field waveforms of atmospheric discharges and particle fluxes on the millisecond time scale^22^. The TGE data is related to the atmospheric discharge measurements provided by networks of antennas and near surface electric field sensors located nearby particle detectors. Fast electronics provides GPS stamp on each registered event. The TGE events and lightning occurrences also are supported by measurements of the World Wide Lightning Location Network (WWLLN) and 30 Hz cameras making photos of the skies above Aragats during lightning occurrences.Detailed information on particle detectors used is available from the supplementary material.

## TGE abruptly terminated by lightning: 28 July TGE

Spring-Summer on Aragats is the time of very strong thunderstorm activity. On 28 July 2016 large disturbances of the near surface electrostatic field began at 12:00 UT. A severe storm started ~1.5 hours later with numerous positive and negative lightning flashes. The atmospheric electricity sign convention (a downward-directed electric field change vector is considered positive) is used throughout this paper. The field change for negative lightning that lowers negative charge overhead is positive.

**Figure 1:**
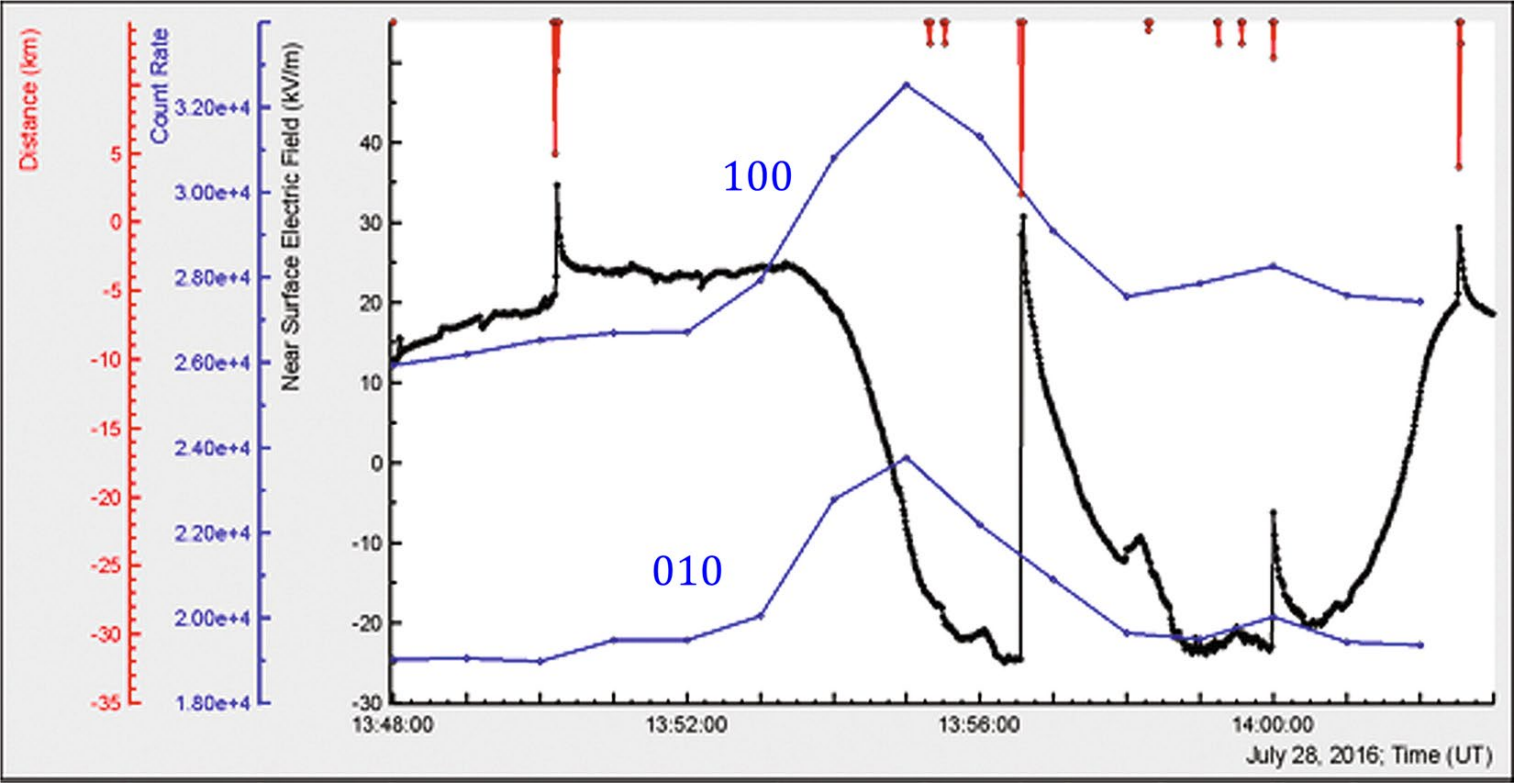
One-minute time-series of STAND1 detector: the count rate combinations 100 and 010 correspond to signals only from upper and middle scintillation detectors, respectively. Flux enhancement at 13:55–13:56 UT is ~34% (44σ). Electric field decreased from +25 kV/m at 13:53:25 UT to −24 kV/m at 13:55:25 UT. Distances to 4 nearby lightning flashes measured by the same EFM-100 device are 4.8, 1.9, 11.7, and 3.8 km from left to right, respectively. Other 6 lightning flashes shown in the top of picture occurred at distances more than 10 km.

At 13:53 UT electrostatic field started to decrease; the same minutes all particle detectors located at Aragats station register enhancement of particle flux (TGE, Fig. 1). A strong lightning at 13:56:34 UT terminated TGE. During 5 minutes of the large flux atmospheric pressure was 693 mbar; relative humidity – 90%, wind speed 2–3 m/sec from ~340° N direction, temperature ~5.9 C°, no rain was registered. Solar radiation was very low, reaching minimum of ~11 W/m^2^ during TGE event. During the maximal flux of TGE the electrostatic field was in negative domain reaching −24 kV/m at 13:56 UT.

We start the analysis of the 28 July TGE event by examining the pattern of correlated measurements of one-minute time series of the STAND1 detector and the disturbances of the electrostatic field at detector site (see Fig. 1). From this initial pattern we can observe:

- Direct relation of TGE to negative electrostatic field measured at detector site;
- Presence of the negative nearby lightning during TGE; the amplitude of electrostatic field change exceeds 50 kV/m;
- Start and rise of TGE occurred before the lightning flash;

Comparison of 2 time series of STAND1 detector allows to roughly estimate the fraction of electrons in TGE: if amplitudes of peaks of “010” time series (mostly gamma rays) and “100” (gamma rays and low energy electrons) are more or less coinciding - the fraction of electrons is minimal.

**Figure 2:**
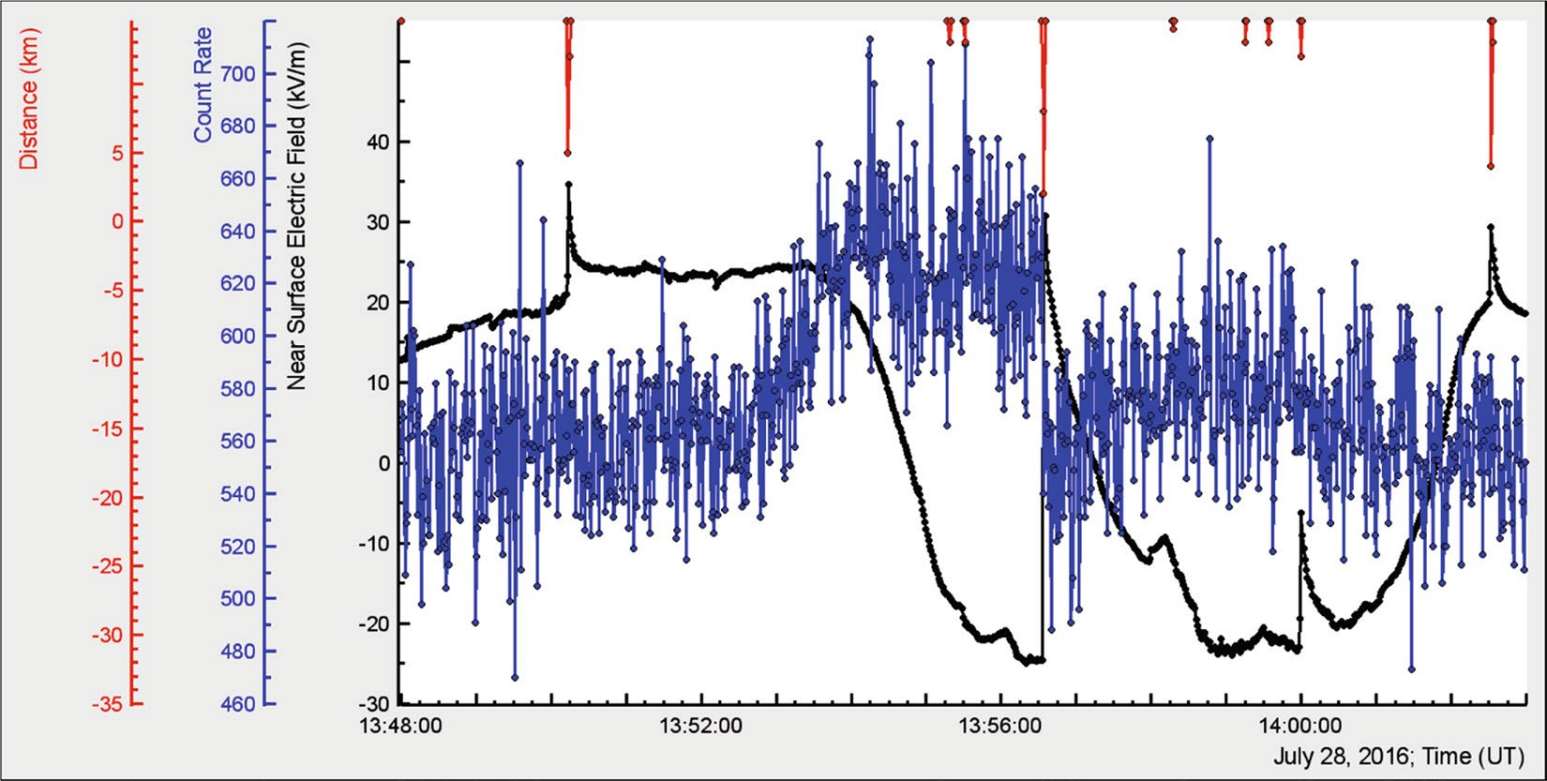
One second time series of 1 cm thick scintillator of STAND1 detector located nearby MAKET experimental hall. Negative lightning abruptly terminated TGE.

Examining the second pattern with one-second time series (Fig. 2) of the outdoors 1-cm thick plastic scintillator we see in much more details the fine structure of TGE:

- The particle flux from the cloud is not uniform on second time scales, exhibiting several 1-second spikes and deeps during 2 minutes of maximal flux;
- Negative lightning abruptly terminates TGE; in one second starting at 13:56:33 the flux decreases from 654 to 541, that is, by 17.2%. After abrupt termination the flux starts to rise again although does not reach previous maximum;
- The electrostatic field recovery needs much more time ~2 minutes.

**Figure 3:**
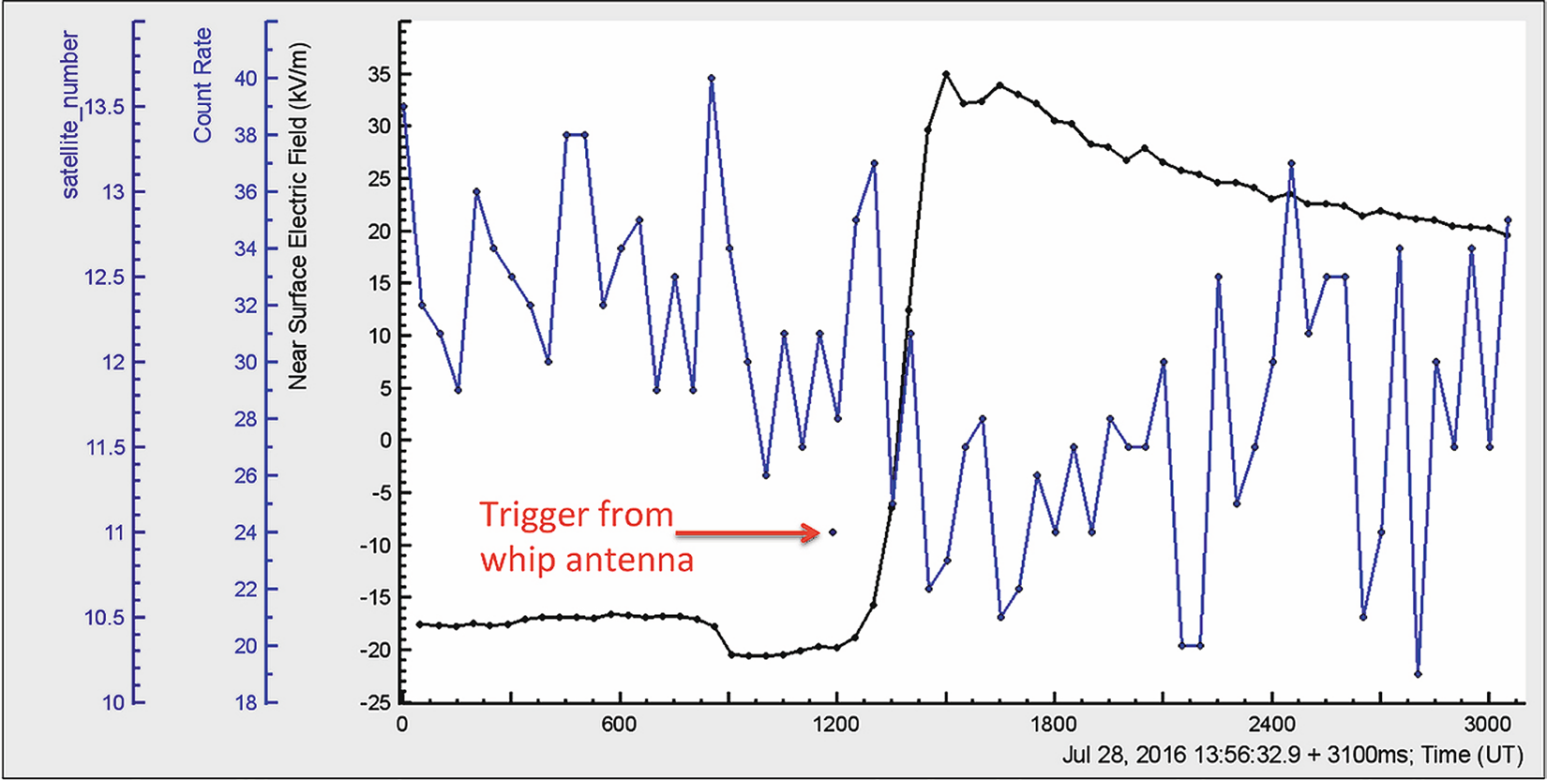
50 ms time series of MAKET upper 1-cm thick scintillator count rate and electrostatic field. The time of trigger is denoted by a point occurred at 13:56:34.087 UT (calculated by 11 GPS satellites). WWLLN registered lightning at 13:56:34.087 UT. The electric field starts to rise ~50 ms after trigger, reaching maximum ~200 ms later; the amplitude of the electric field change was ~48.6 kV/m. Particle flux starts to decline at 11:56:34.2 UT.

Next data pattern (Fig. 3) includes 50 ms time series of the count rate of 1 cm thick plastic scintillator and disturbances of the electrostatic field, as well as the time of the trigger (shown by an arrow) produced by the signal from a commercial MFJ-1022 active whip antenna. After the trigger signal, which denotes the start of significant electromagnetic emission from lightning, digital oscilloscope generates a file with electric field waveforms produced by lightning (1 s record length, including 200 ms before trigger and 800 ms after trigger).

From this data analysis we can get the following information:

- The exact time of lightning flash reported by WWLLN is 13:56:34.087 UT (same as for the trigger);
- Particle count rate decline occurs after the trigger at 11:56:34.2 UT simultaneously with start of rearrangement of electrostatic field;
- TGE decay started simultaneously with an abrupt increase of the near-surface electrostatic field. Therefore, the termination of TGE is directly connected to the rearranging of charged structures in the thundercloud, which is governed by lightning.

From the presented above patterns (Figs 1–3), we can see how the RB/RREA process in thunderclouds is related to the disturbances of the electric field (including lightning flash) above particle detectors:

- Particle flux start to rise on declining of the electrostatic field and TGE reaches maximum on the minimum of the field strength;
- On the rising phase of TGE no lightning occurred before the particle flux was abruptly terminated by a strong negative lightning stroke. Lightning flash rise time was ~100 ms, recovery ~2 minutes;
- The rearrangement of the electric field in the cloud and particle flux decline occurred the same time after lightning stroke.

To gain more insights into the avalanche processes in the cloud we measured the intensities of electron and gamma ray fluxes, as well as energy spectra of the gamma rays available from the variety of spectrometers on Aragats. To select TGEs with small electron contamination we used thick plastic scintillators fully shielded by thin scintillators vetoing charge flux (see CUBE detector description in the attachment). Correcting the fluxes due to possible miscount of gamma rays and electrons caused by not 100% detection efficiency of the scintillators according to techniques described in ref. 8 we readily come to the intensities shown in Table 1. The intensities were recovered separately for 2 vertically stacked 20-cm thick plastic scintillators.

**Table 1:** Recovered intensities of the electrons and gamma rays of TGE for the upper and lower 20 cm thick scintillators.

28 July 2016	Upper scintillator			Bottom scintillator		
	e intensity (1/m^2^min)	γ intensity (1/m^2^min)	e/γ (%)	e intensity (1/m^2^min)	γ intensity (1/m^2^min)	e/γ (%)
13:52–13:53	69	1123	6^*^	55	425	13*
13:53–13:54	408	3363	12^*^	0	2172	0
13:54–13:55	460	23328	2.0	0	3524	—
13:55–13:56	992	15608	6.4	760	9532	8.0
13:56–13:57	92	8540	1.1	0	1500	—
13:57–13:58	0	772	—	0	460	—

*For the low intensities the estimate of electron fraction is unstable.

**Figure 4:**
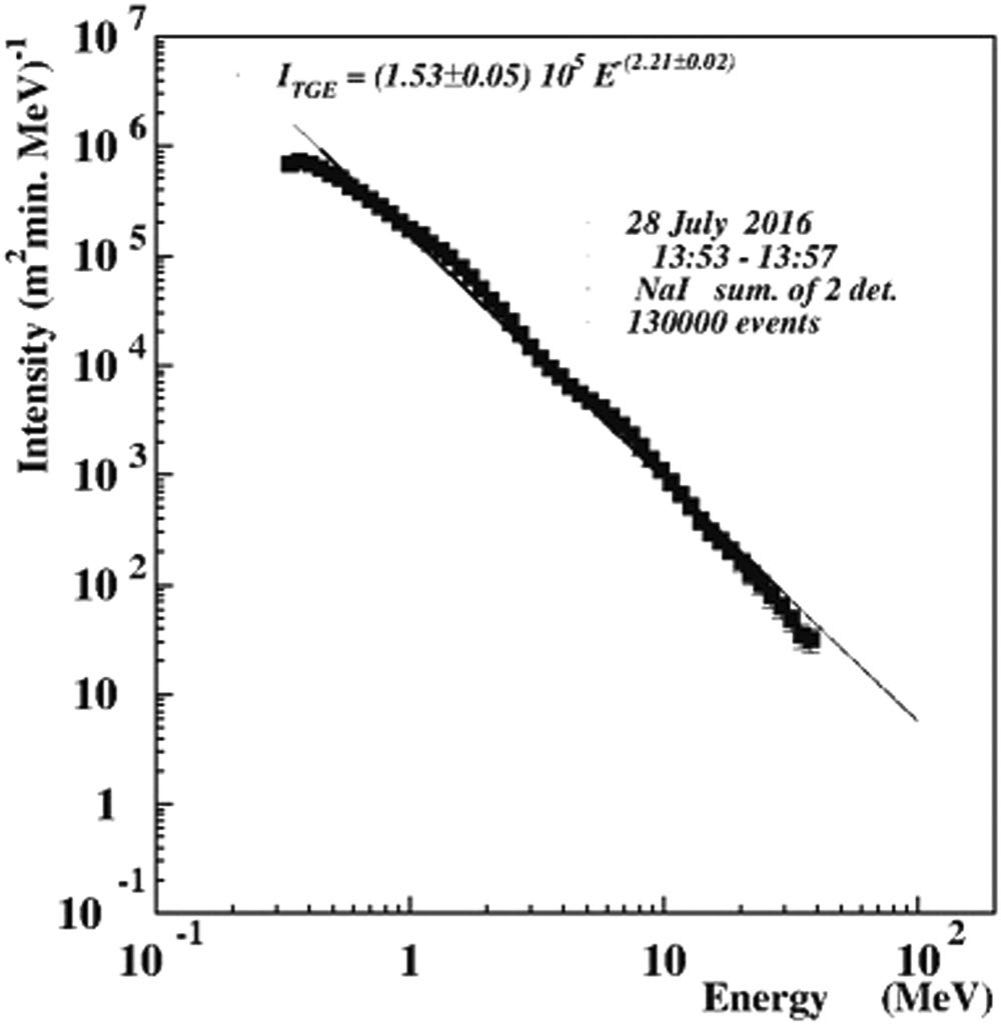
Energy spectrum of TGE exteneded up to 30 MeV measured by 2 NaI spectrometers during 4 minutes of TGE.

In Table 1 we show a large flux of the high-energy particles at 13:54–13:57 UT; at 13:58 UT the flux abruptly declines. The high fraction of electrons in the lower thick scintillator is an indication of the intense RB/RREA process in the cloud above the detector. The ultimate check of the presence of the high-energy electrons and gamma rays in TGE is the energy spectra recovered by the network of NaI spectrometers extended up to 30 MeV (see Fig. 4).

The sizes of NaI crystals are rather small (12 × 12 × 24 cm), and to have a statistically significant number of events in the highest energy bins of histogram we need to keep data collecting time not smaller than 1 minute. With 1 m^2^ area 60 cm thick plastic scintillator we can lower the collecting time down to 20 sec and register rather small intensities, corresponding to highest energies. However, the 60 cm thick plastic scintillator comprises only 1.4 radiation lengths (RL); the thickness of the NaI crystal corresponds to 4.6 RL. Therefore, for the 60 cm thick scintillator we present only energy release spectra and do not recover energy spectra.

**Figure 5:**
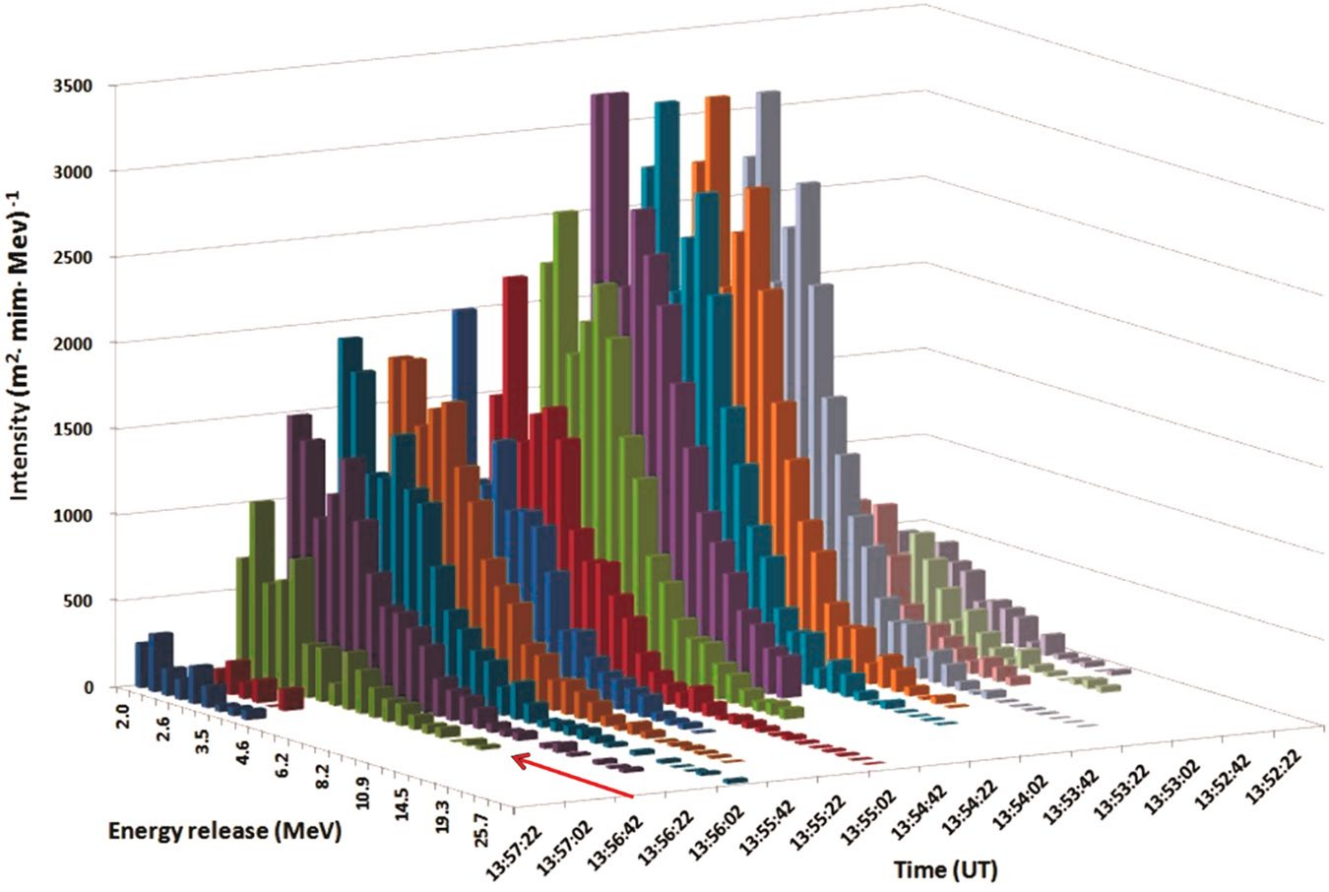
3-dimensional histogram of the energy release measured by the 60-cm thick plastic scintillator during the time interval from 13:52:22 UT to 13:57:22 UT. The red arrow shows lightning occurred at 13:56:34.

From Fig. 5 it is apparent that maximal energy particles had illuminated particle detectors randomly in the time span of 13:53–13:56 UT. Before the lightning occurred at 13:56:34 UT the energy release spectra were extended up to 20 MeV and more. After lightning, the intensity and maximal energy of gamma rays significantly decrease. In ref. 5 we have demonstrated that TGE is a superposition of multiple runaway cascades initiated by the CR electron randomly entering strong electric field region in the cloud. We name such a cascade Extensive Cloud Showers (ECSs); authors of^23^ name it Micro Runway Breakdowns – MRB. On the minute time scale (Fig. 1) we see a rather smoothed pattern of the TGE; when we turn to 1-second time scale (Fig. 2) we see random fluctuations of the TGE intensity and recognize corresponding changes in the maximal energy (Fig. 5). RB/RREA is a random process dependent on the fast changing distribution of charge centers in the cloud, on atmospheric discharges, wind speed and other.

From the recovered overall energy spectrum of TGE (Fig. 4) and 20-second energy release spectra (Fig. 5) showing the dynamic of changing particle fluxes we can conclude that:

- Maximal intensity of the TGE was observed at 13:54–13:56 UT;
- Recovered energy spectrum by the NaI crystal network demonstrate high-energy particle tail up to 30 MeV;
- Observations of the energy release histograms with 60 cm thick plastic scintillator outline different episodes of the high-energy emission;
- In the energy spectra measured after lightning, at 13:56:42–13:57:22 UT intensities abruptly declined and the highest energy particles vanished.
- The RB/RREE process was developed in the thundercloud and high-energy particles illuminate detectors in the time span of 13:54–13:56:34 UT, before the lightning stroke.

**Figure 6:**
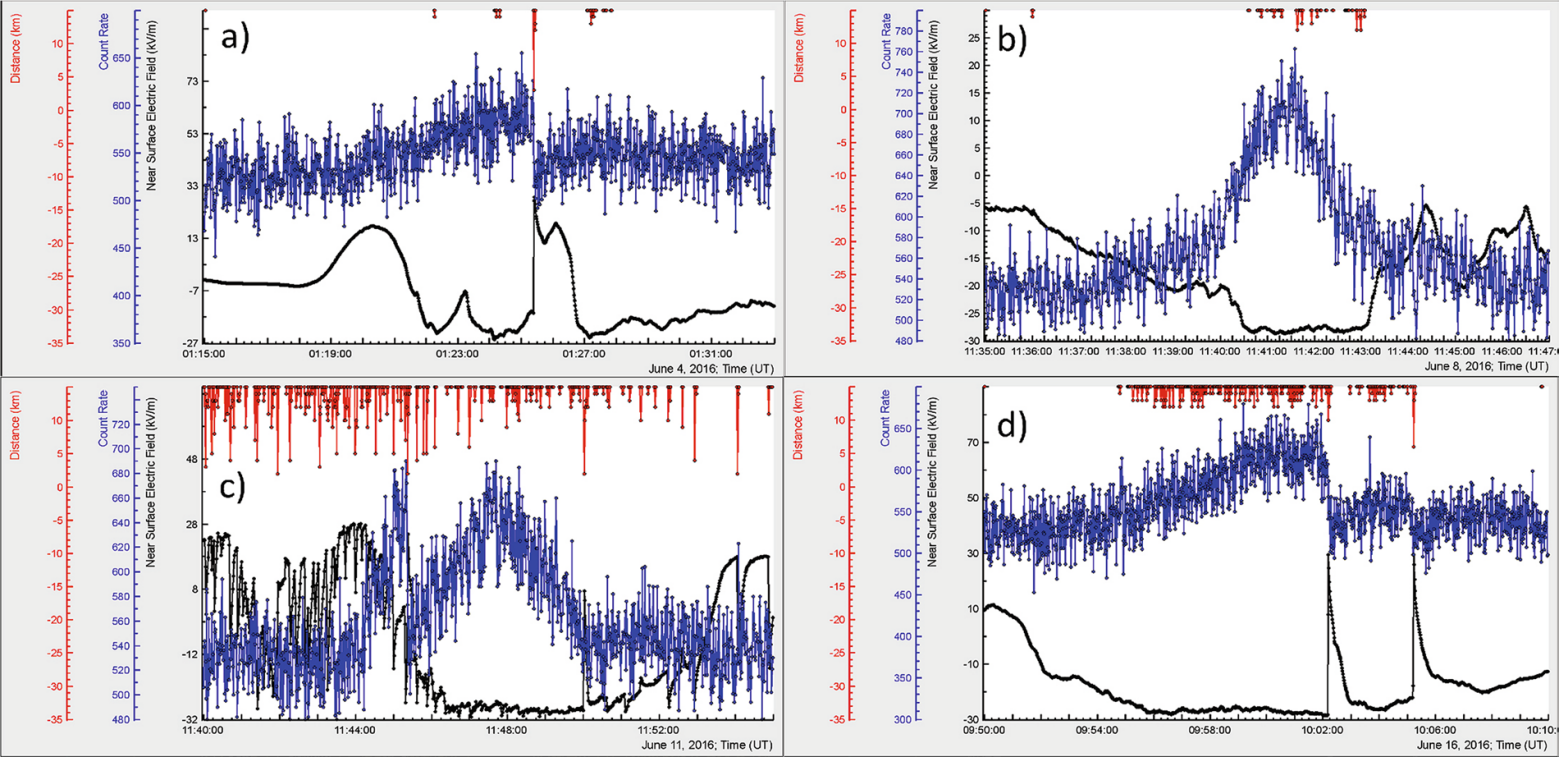
June TGE events abruptly terminated by nearby lightning (**a**,**c**,**d**) and – not terminated (**b**). The upper 1-cm thick plastic scintillator of STAND1 detector located nearby MAKET experimental hall measures one-second count rate. Electrostatic field and distance to lightning are measured and estimated by the EFM 100 electric mill located nearby GAMMA array.

We analyzed largest TGE events of spring – summer 2016 mostly abruptly terminated by the lightning discharge, see Fig. 6 and Table 2 (TGE data is available from the site http://www.crd.yerphi.am/adei/). In Fig. 6 we show the TGE’s occurred in June, the stormiest month of 2016. As we see from Fig. 6 these TGEs share the common features of July 28 TGE. Particle flux increased when the electrostatic field is in the negative domain; nearby lightning abruptly terminated TGE, after lightning flash the particle flux abruptly terminated and again started to increase (Fig. 6a,c,d). The TGE in Fig. 6b smoothly decays after reaching maximum; distant lightning flashes, which occurred more than 10 km apart, do not terminate it.

**Table 2:** Characteristics of the TGE events detected in Spring-Summer 2016.

Date	Start of TGE (UT) and el. field value kV/m	Time of maximum (UT) and el. field kV/m	TGE significance %/N of sigma	*L*.	time (min)	Drop of flux %	Surge of el. field kV/m	EFM Dist.km	*Max. En.MeV*
28/04	18:19	18:23	20.6/22.6	2	4	13.2	60	1.9	50
	−6.3	−13	18.5/20.5		5:30	15	71	1.9	
4/05	18:57	19:04	45.7/56	2	7	27	54.5	1.9	40
	−2.4	−12.3			8:30	14	52.5	2.9	
10/05	14:07	14:14	24/32.6	2	7	29	58.5	7.9	40
	−26	−29	16.5/22.3			15	50.1	7.9	
12/05	13:40/−15	13:47/−29	13/27		7			10.8	50
15/5	02.21	02:27	20.6/17	2	6	17.7	56	9.7	40
	15	−27			7:20	16.5	57.7	4	
4/6	01:17/−3.3	01:25/−21.3	17.9/17.6	1	8	15.9	43.1	3	60
8/6	11:37/−15	11:42/−26.5	32.1/37		5			13	50
11/6	11:38	11:48	26.2/36.7		10	19.2	51.6	1.8	10
	41	−27			15	9.5	36.1	1.9	
16/6	1:53	10:02	18/28	2	9	25	57	9.6	40
	−15	−26			12	11	53.3	5.8	
28/7	13:50	13:55	34/44	1	5	14.5	38.6	1.8	30
	4	−16							

In Table 2 we show all large TGEs of 2016. In the first column of Table 2 we post the date of the event; in second and third columns - the time of TGE start and time of reaching maximal flux and, below in the same cells - corresponding values of the electrostatic field strength; in the fourth column - TGE significance in percent of increase related to pre-TGE count rate and in number of standard deviations (the “100” combination of the STAND1 detector located nearby MAKET experimental hall was chosen as reference count rate); in the fifth– number of lightnings terminating TGE; in the sixth column - drop of TGE flux after lightning (if any); in the seventh – surge of near surface electrostatic field after lightning; in the eighth – distance to lightning estimated by EFM-100 electric mill; in the ninth – total duration of TGE; and in the tenth - maximal energy of differential energy spectra estimated by the network of NaI spectrometers. If two or more peaks are observed in the TGE we show in the Table the time of maximum and corresponding electrostatic field only for the first (usually largest) peak.

One-minute time series of the particle detectors with low energy threshold demonstrate huge enhancements equivalent to tens of standard deviations. The differential energy spectrum of gamma rays extends up to 40 MeV and beyond proving intense RREA process in the thundercloud above the detector site. The strong negative lightning is seen as an abrupt increase of the near-surface electrostatic field with an amplitude of ~50 kV/m and more; all observed lightning discharges that terminated TGE events at Aragats lowered the negative charge overhead. Only nearby lightning flashes (within 10 km) terminated TGE. Only in 2 events from 10, we do not register an abrupt decline of TGE flux and nearest lightning flashes for these events were at distance more than 10 km. In 8 events from 10 nearby lightning abruptly terminated TGE.

## Discussion and Conclusions

Several severe storms were accompanied with intense particle fluxes observed by facilities of Aragats Space environmental Center. By examining TGE events we see that before lightning the intensity of the RB/RREA reaches maximal value and maximal energy of avalanche particles reach 40 MeV and more. After lightning, we detect an abrupt decrease of particle flux caused by the removal of high-energy particles. All these processes occurred within few hundreds of millisecond. All observed TGE-terminating lightning flashes lowered negative charge overhead. Therefore, we can connect by causal relation the RB/RREA process and the lightning initiation, i.e. RB/RREA process in the thundercloud serves as a trigger to the negative lightning.

**Figure 7:**
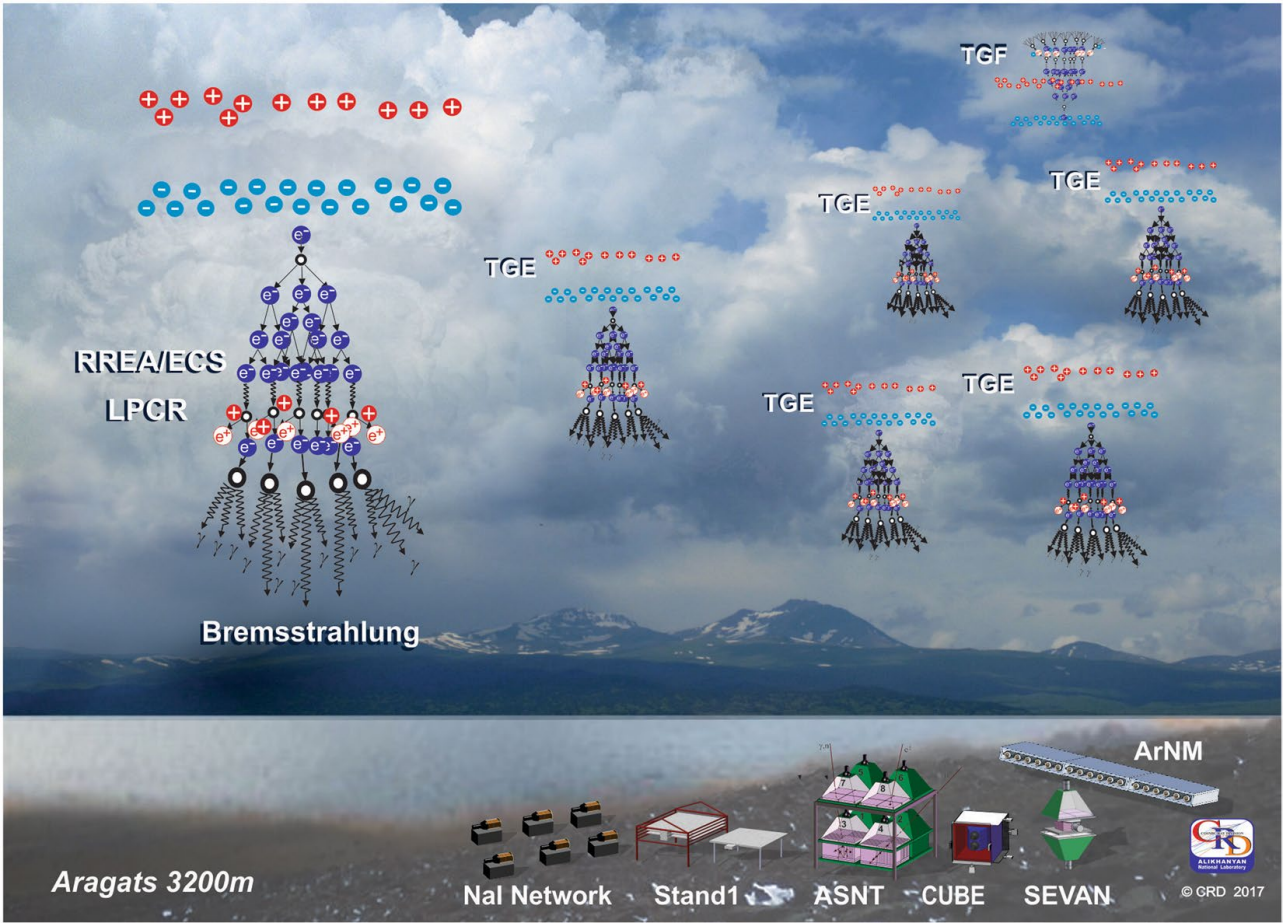
Cartoon illustrated TGE and TGF initiation above Aragats mountain. This figure is not covered by the CC BY licence. [Credits to Cosmic Ray Division (CRD)]. All rights reserved, used with permission.

For our conclusion on the lightning initiation, we use the only small subsample of lightning flashes in the observed storm (flashes terminating particle flux). TGE is a rather rare transient process depending on the coincidence of several random parameters of the electrified atmosphere. The size of the radiation-emitting region in thundercloud is 500–1000 m^24, 25^ and only by chance this region for several minutes is positioned above the particle detectors. Our particle detectors are not positioned in some specially selected area, as in beam experiments with man-made accelerators. Only by chance the strength of electric field can exceed the RB/RREA initiation threshold in the cloud just above this region. Another key parameter is a vertical extension of the electric field, which must be long enough to provide necessary potential drop. Thundercloud, as well, should be low enough above earth’s surface; in other cases, the electron-gamma ray avalanche will be faded in the air. However, if RREA initiation conditions are fulfilled somewhere in the huge thundercloud, RREA process will be unleashed (see Fig. 7).

The authors of^26^ after examining of 23 thunderstorm electric field soundings suggest that lightning may occur whenever the electric field exceeds the “breakeven” field (for field strengths greater than the breakeven field, an energetic electron’s energy increases with time).

The group from Langmuir Laboratory in central New Mexico during balloon flights on 3 July 1999 measured the maximal field of 1.86 kV/cm (130% of the threshold for a runaway process) at 5.77 km altitude just before nearby lightning flashes^27^. Authors conclude that RB/RREA avalanches have limited the magnitude of the electric field inside storms and initiated lightning flashes.

Thus, both our measurements based on TGE detection terminated by a lightning flash and *in situ* measurements of an intracloud electric field along with lightning discharges prove that RB/RREA is an apparent mechanism for the initiation of the negative lightning flashes.

Therefore, the following scenario of the lightning initiation can be suggested:

1. In the thunderstorm cell randomly emerge extended regions of enhanced electric field with strength above the breakeven limit (for instance electric field of 1.8 kV/cm on 4–6 km heights and ~1 km extension); these regions are randomly distributed in the cloud and are continuously moved due to rather strong wind on 4–8 km heights.
2. At the same heights, the flux of secondary cosmic ray (CR) electrons with energies appropriate for the runaway regime (100 KeV − 2 MeV) is significantly high - several thousands of particles per (s · m^2^). These CR seed electrons entering high electric field regions unleash the RB/RREA process producing large particle fluxes.
3. Intense particle flux create a system of the random clusters of ionization in a huge 3-dimension storm cell. Due to some, yet unspecified stochastic mechanism (for an example of such a mechanizm, see ref. 28) in some place in the cloud a discharge occurred, stopping TGE and initiated lightning.
4. Due to working charging machine in the cloud at another time in another place points 1–3 will be repeated as a storm prolonged (see Fig. 7).

## Electronic supplementary material

Instrumentaion
