# Supplementary material for: On the initiation of lightning in thunderclouds
Source: Sci Rep. 2017 May 2;7:1371. doi: 10.1038/s41598-017-01288-0 (PMC5430999; doi:10.1038/s41598-017-01288-0)
Supplement: Supplementary file 1 — Instrumentaion [file 41598_2017_1288_MOESM1_ESM.pdf]

On the initiation of lightning in thunderclouds

(Instrumentation, Supplementary information)

Ashot Chilingarian[1,2], Suren Chilingaryan[1], Tigran Karapetyan[1], Lev Kozliner[1], Yeghia Khanikyants[1], Gagik Hovsepyan[1], David Pokhsraryan[1] and Suren Soghomonyan[1]

[1]Yerevan Physics Institute, 2 Alikhanyan Brothers, 0036, Yerevan, Armenia
[2]National Research Nuclear University MEPhI (Moscow Engineering Physics Institute), Moscow 115409, Russian Federation

The "STAND1" detector is comprised of three layers of 1-cm-thick, 1-m^2 sensitive area scintillators fabricated by the High Energy Physics Institute, Serpukhov, Russian Federation; see Fig. 1. The light from the scintillator through optical spectrum-shifter fibers is reradiated to the long- wavelength region and passed to the photomultiplier (PMT FEU-115M). The maximum of luminescence is emitted at the 420-nm wavelength, with a luminescence time of about 2.3 ns. The STAND1 detector is tuned by changing the high voltage applied to the PMT and by setting the thresholds for the shaper-discriminator. The discrimination level is chosen to guarantee both high efficiency of signal detection and maximal suppression of photomultiplier noise.

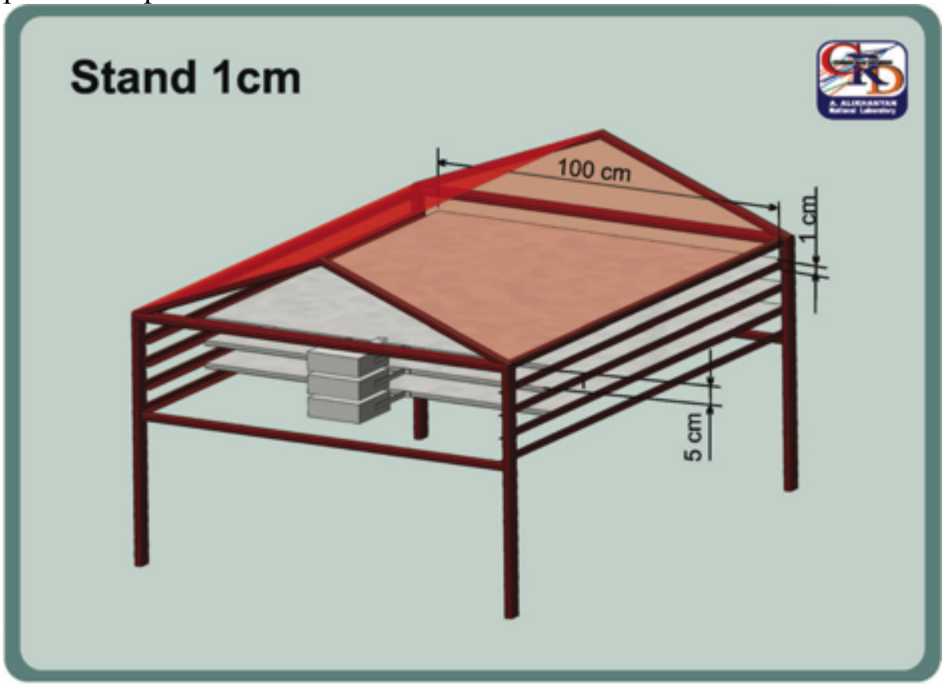

Figure 1. STAND1 detector consisting of three layers of 1-cm- thick scintillators.

Special experimental facilities were designed and installed at Aragats in order to separate electron and gamma ray fluxes. Two 20-cm-thick plastic scintillators are surrounded by 1-cm-thick molded plastic scintillators (see Fig. 2). Thick scintillators

detect charged flux with a very high efficiency (~99%); they can also detect neutral flux with an efficiency of ~20%. Thin scintillators also detect charged flux with very high efficiency (~99%), though the efficiency of detecting neutral flux is highly suppressed and equals 1%–2%. Thus, using the coincidences technique, it is possible to purify the neutral flux detected by inside scintillators, rejecting the charged flux by the veto signals from surrounding thin scintillators. The calibration of the cube detector proves that the veto system (preventing the counting signal in the thick scintillator if there is a signal in at least one of the six surrounding thin scintillators) can reject 98% of the charged flux. The histograms of the energy deposits in the two inner thick scintillators are stored every minute. The one-minute count rates of the surrounding 6 scintillators are measured and stored as well.

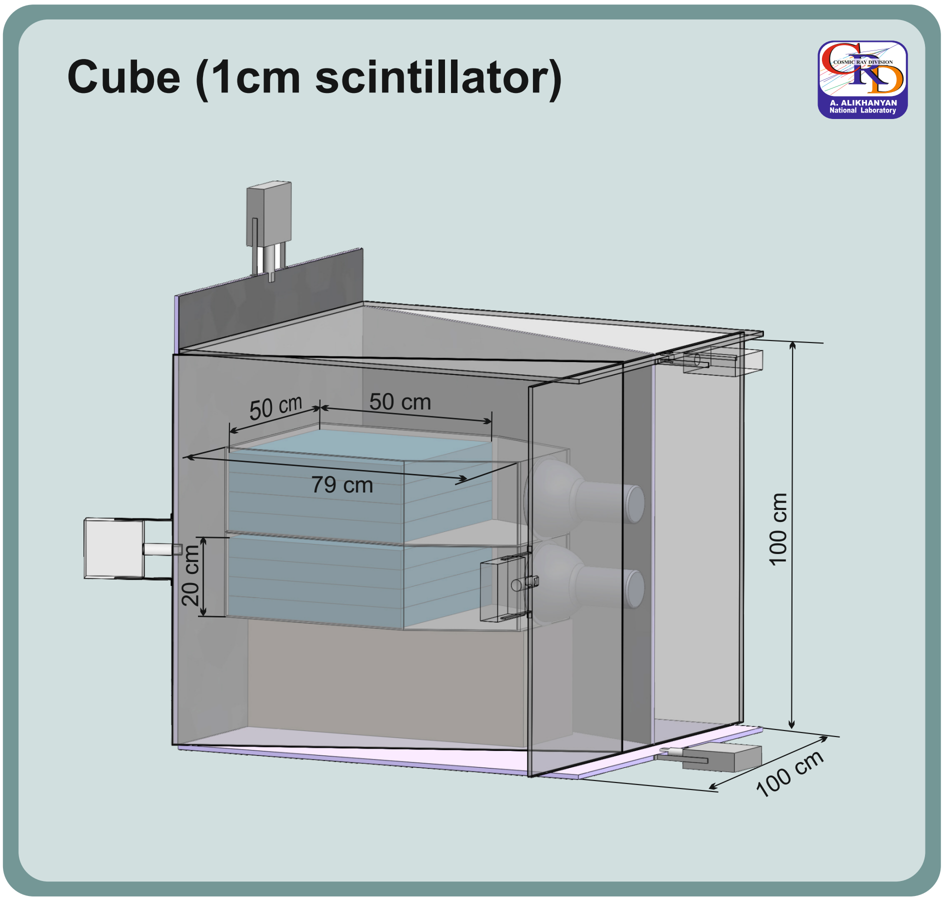

Figure 2. CUBE detector. Six 1-cm thick scintillators are used as a veto system for the charged particles. Inner two 20 cm thick scintillators detect both charged and neutral fluxes.

The detector network measuring particle energy consists of 4 NaI crystal scintillators packed in a sealed 3-mm- thick aluminum housing. The NaI crystal is coated by 0.5 cm of magnesium oxide (MgO) by all sides (because the crystal is hygroscopic) with a transparent window directed to the photo-cathode of an FEU-49 PMT, see Fig. 3. The large cathode of PMT (15-cm diameter) provides a good light collection. The spectral sensitivity range of FEU-49 is 300–850 nm, which covers the spectrum of the light emitted by NaI(Tl). The

sensitive area of each NaI crystal is ~0.0348 m^2, the total area of the four crystals is ~0.14 m^2, and the gamma-ray detection efficiency is ~80%. A logarithmic analog-digit converter (LADC) is used for the coding of PM signals. Calibration of LADC and code-energy conversion was made by detecting the peak from exposed ^{137}Cs isotope emitting 662 keV gamma rays and by the high-energy muon peak (55 MeV) in the histogram of energy releases in the NaI crystal. The PMT high voltage was tuned to cover both structures (peaks) in the histogram of LADC output signals (codes) and to ensure linearity of LADC in the energy region of 0.4–60 MeV. The count rate of a particle detector depends on the chosen energy threshold of the shaper-discriminator, the size of the detector, and the amount of matter above it. The inherent discrepancy of the parameters of PMTs also can add ~15 % difference to the particle detector count rates. A significant amount of substance above the sensitive volume of NaI crystals (0.7 mm of roof tilt, 3 mm of aluminum, and 5 mm of MgO) prevents electrons with energy lower than ~3 MeV from entering the sensitive volume of the detector. Thus, the network of NaI spectrometers below 4 MeV can detect gamma rays only.

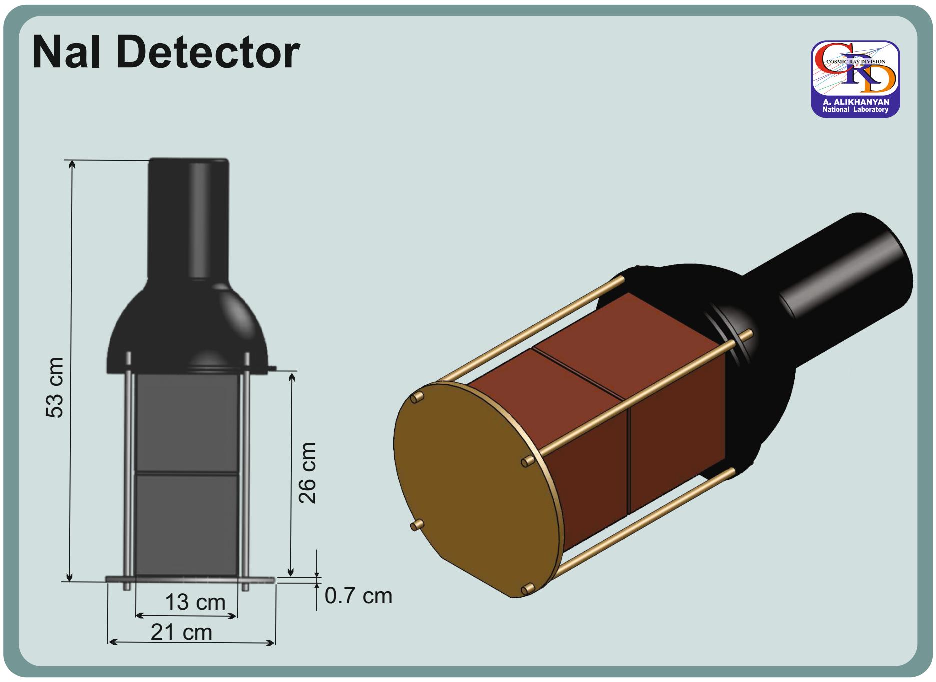

Figure 3. NaI(Tl) crystal assembly.

The Aragats Solar Neutron Telescope (ASNT, previously intended to measure neutrons coming from violent solar flares) is formed from 4 separate identical modules, as shown in Fig. 4. Each module consists of forty 50 x 50 x 5 cm^3 scintillator slabs stacked vertically on a 100 x 100 x 10 cm^3 plastic scintillator slab. Scintillators are finely polished to provide good optical contact of the assembly. The slab assembly is covered by the white paper from the sides and bottom and firmly kept together with special belts. The total thickness of the assembly is 60 cm. Four scintillators of 100 x 100 x 5 cm^3 each are located above the thick scintillator assembly to indicate charged particle traversal and separate the neutral particles by "vetoing" charged particles (the probability for the neutral particle to give a signal in 5 cm thick scintillator is much lower than in 60 cm thick scintillator). A scintillator light capture cone and Photo Multiplier Tube (PMT) are located on the top of the scintillator housings.

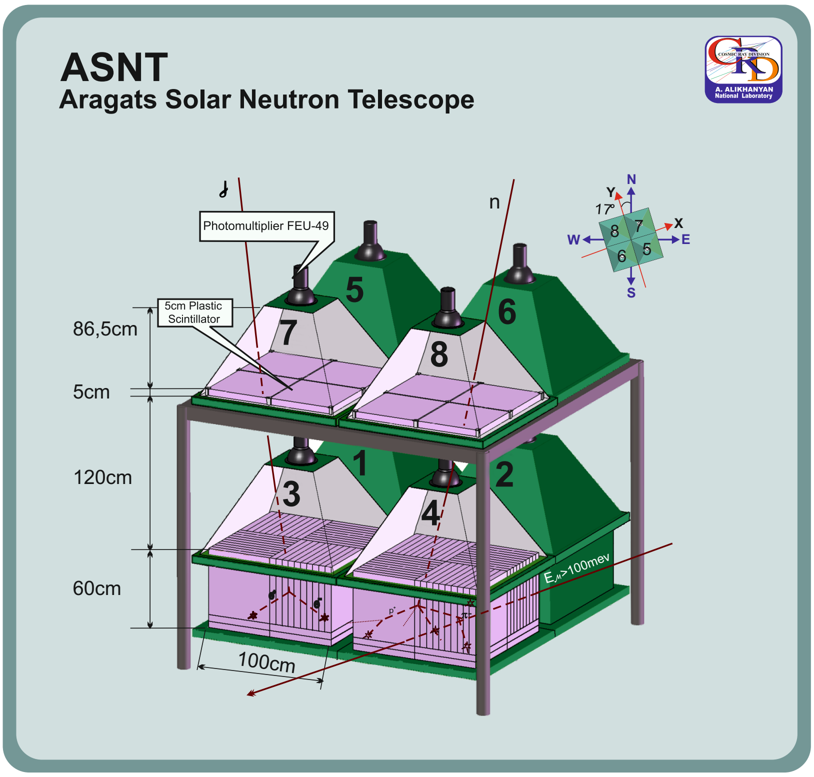

Figure 4. Assembly of ASNT with the enumeration of 8 scintillators and orientation of detector axes relative to the North direction.

The main ASNT trigger reads and stores the analog signals (PMT outputs) from all 8 channels if at least one channel reports a signal above threshold. The frequency of triggers is ~ 4 KHz due to incident Secondary cosmic rays (SCR) – products of the interaction of galactic cosmic rays with atmosphere; on 3200 m height on Aragats, the intensity of SCR is ~ 500 /m^2/sec. The flux of particles from thundercloud (TGE) can be 5 times larger than SCR (background) intensity.

The list of available information from ASNT is as follows:

1. 2 second time series of count rates of all 8 channels of ASNT (the integration time of the scintillator counts is 2 seconds);
2. Count rates of particles arriving from the different incident directions: 16 possible coincidences of 4 upper and 4 bottom scintillators;
3. Count rates of the 8 special coincidences, for instance, 1 signal from the upper scintillators and 1 signal from the lower ones, or no signals in upper, and more than 1 signal in the lower, etc.;
4. Estimates of the variances of count rates of each ASNT channel, variances are calculated by 12 five-second counts, i.e. in a minute 12 times (each with 5 sec integration time) all channel counts are stored; then with stored values the means and variances are calculated;
5. 8 x 8 correlation matrix of ASNT channels calculated by five-second count rates in 1 minute; with same stored values of the 5-sec time series each minute the correlation matrix is calculated to monitor possible cross-talk of channels;
6. Each minute (after 07.2012, each 20 second) the histograms of the energy releases in all 8 channels of ASNT are stored;

7. The same as in the previous point, but only for particles that dos not registered in the upper layer (veto on charged particles to select samples enriched by neutral particles);

A 52 cm diameter circular flat-plate antenna was used to record the wideband electric field waveforms produced by lightning flashes. The antenna was followed by a passive integrator the output of which was directly connected with a 60 cm double-shielded coaxial cable to a Picoscope 5244B digitizing oscilloscope. The oscilloscope was triggered by the signal from a commercial MFJ-1022 active whip antenna that covers a frequency range of 300 kHz to 200 MHz. The record length was 1 sec including 200 ms pre-trigger time and 800ms post-trigger time. The sampling frequency was 25 MS/s, and the amplitude resolution was 8 bit. The trigger-out pulse of the oscilloscope was relayed to the NI myRIO board which produced the GPS time stamp of the record.

The near-surface electrostatic field changes were measured by a network of six field mills (Boltek EFM-100), four of which were placed in Aragats station, one in Nor Amberd station at a distance of 12.8 km from Aragats, and another one in Yerevan station at a distance of 39.1 km from Aragats. The electrostatic field measurements were taken with an interval of 50 ms. Lightning optical image is captured by a video camera at a frame rate of 30 frames/s. We used also data from the World Wide Lightning Location Network (WWLLN), which detects very low frequency (VLF, 3-30 kHz) emissions from lightning. Boltek’s EFM-100 electric mill also provides estimates of the distance to lightning.
